# Yield optimization, microbial load analysis, and sensory evaluation of mungbean (*Vigna radiata* L.), lentil (*Lens culinaris* subsp. *culinaris*), and Indian mustard (*Brassica juncea* L.) microgreens grown under greenhouse conditions

**DOI:** 10.1371/journal.pone.0268085

**Published:** 2022-05-24

**Authors:** Priti, Seema Sangwan, Bharti Kukreja, Gyan Prakash Mishra, Harsh Kumar Dikshit, Ajeet Singh, Muraleedhar Aski, Atul Kumar, Yashpal Taak, Tsering Stobdan, Shouvik Das, Ranjeet R. Kumar, Devendra Kumar Yadava, Shelly Praveen, Shiv Kumar, Ramakrishnan M. Nair

**Affiliations:** 1 Division of Genetics, ICAR-Indian Agricultural Research Institute, New Delhi, India; 2 Division of Microbiology, ICAR-Indian Agricultural Research Institute, New Delhi, India; 3 Division of Biochemistry, ICAR-Indian Agricultural Research Institute, New Delhi, India; 4 Division of Seed Science & Technology, ICAR-Indian Agricultural Research Institute, New Delhi, India; 5 DRDO-Defence Institute of High Altitude Research, Leh-Ladakh, India; 6 Biodiversity and Integrated Gene Management Program, ICARDA, Rabat, Morocco; 7 World Vegetable Center, South Asia, ICRISAT Campus Patancheru, Hyderabad, India; Purdue University, UNITED STATES

## Abstract

Microgreens have been used for raw consumption and are generally viewed as healthy food. This study aimed to optimize the yield parameters, shelf life, sensory evaluation and characterization of total aerobic bacteria (TAB), yeast and mold (Y&M), *Escherichia coli*, *Salmonella* spp., and *Listeria* spp. incidence in mungbean (*Vigna radiata* (L.) Wilczek), lentil (*Lens culinaris* Medikus subsp. *culinaris*), and Indian mustard (*Brassica juncea* (L.) Czern & Coss.) microgreens. In mungbean and lentil, seeding-density of three seed/cm^2^, while in Indian mustard, eight seed/cm^2^ were recorded as optimum. The optimal time to harvest mungbean, Indian mustard, and lentil microgreens were found as 7^th^, 8^th^, and 9^th^ day after sowing, respectively. Interestingly, seed size was found highly correlated with the overall yield in both mungbeans (r^2^ = .73) and lentils (r^2^ = .78), whereas no such relationship has been recorded for Indian mustard microgreens. The target pathogenic bacteria such as *Salmonella* spp. and *Listeria* spp. were not detected; while TAB, Y&M, *Shigella* spp., and *E*. *coli* were recorded well within the limit to cause any human illness in the studied microgreens. Washing with double distilled water for two minutes has shown some reduction in the overall microbial load of these microgreens. The results provided evidence that microgreens if grown and stored properly, are generally safe for human consumption. This is the first study from India on the safety of mungbean, lentils, and Indian mustard microgreens.

## Introduction

Microgreens are nutritionally superior food that can be produced from several crops including vegetables, herbs, grains, and some wild species and offer a good option for addressing the problems arising due to rapid urbanization [1]. Microgreens are generally seven to twenty-one days old tender immature greens of 5–10 cm height having three major parts: cotyledonary leaf, stem, and a pair of true leaves [2, 3]. The use of microgreens was reported for the first time during the late 1980s by the chefs working in some restaurants located in San Francisco, California, United States of America (USA) for culinary purposes [4]. The global microgreens market has four broad segments, (i) Green types (Brassicaceae, Asteraceae, Fabaceae or Leguminosae, etc.); (ii) Farm types (outdoor farming, greenhouse farming, vertical farming); (iii) End-uses (food & beverages, cosmetics, etc); and (iv) Region-based (North America, Latin America, Europe, Asia Pacific, Middle East, and Africa) [5].

The USA is a major contributor to the microgreens global market, followed by Canada and Mexico. By geography, North America is leading the microgreens market with a share of nearly 50% in terms of dollar sales in 2019 [6]. The large-scale microgreens farming in the USA and consumption (mostly in the restaurants) are supporting the market in this region [6]. During 2020–2025, the microgreens market at the global level is anticipated to grow at a compound annual growth rate (CAGR) of 7.5–8.0% [7]; while the microgreens market in the USA is projected to register a CAGR of 10.1% [5]. Microgreens are generally produced as an organic product; however, now trend is shifting towards biofortification of microgreens for various minerals including Selenium [8], Fe, and Zn [9]. Microgreens can be comfortably grown irrespective of season in a variety of growing media, depending on the scale of production [3, 10]. Optimization of seeding density and day of harvesting will help in minimizing the microgreens production cost.

Microgreens are usually consumed without heat treatment or decontamination [11] and when compared to sprouts, microbial contamination is not so rampant in microgreens [11, 12]. However, many recalls have happened in the USA and Canada due to *Salmonella* [13, 14], and *Listeria* contamination [15–18]. *Salmonella* spp., *Escherichia coli*, and *Listeria* spp. are the most common bacterial pathogens associated with fresh produce and sprouts [19–23]. The microflora of microgreens is reportedly influenced by the type and composition of growing-medium (soil, peat, vermiculite, or hydroponics) [12, 24, 25], seed-contamination, care taken during harvesting and storage of the microgreens [18].

Being fresh-cut product, microgreens have a relatively very short shelf life, which does vary depending upon the species [26, 27]. The quick post-harvest quality deterioration is also due to their high surface area to volume ratio, delicate leaves, and high respiration rate [28–30]. Immediately after harvest, microgreens can be marketed or should be washed, packed, and stored under cool conditions (1–5 °C) [24, 26]. Thus, various post-harvest treatments such as washing, packaging, and storage conditions become very crucial for extending their shelf life including the sensory qualities of freshly cut microgreens [29–31]. Several sanitizers, including washing of microgreens with tap water, chlorinated water, citric acid, ascorbic acid, and their subsequent storage at 5°C for up to 9 days was reported on Chinese cabbage microgreens [29]. Amongst all the variables, storage temperature is considered as the key factor affecting overall quality including microgreens shelf life [26, 31]. This study was aimed to optimize various yield parameters including seeding density and day of harvesting of microgreens of mungbean, lentil, and Indian mustard. These microgreens were also washed, packed, and stored for a variable duration at refrigeration temperature for shelf life and evaluation of microbial load (TAB, Y&M, *E*. *coli*, *Salmonella* spp., and *Listeria* spp.).

## Material and methods

### Genotypes used and growing conditions

**Fig 1:**
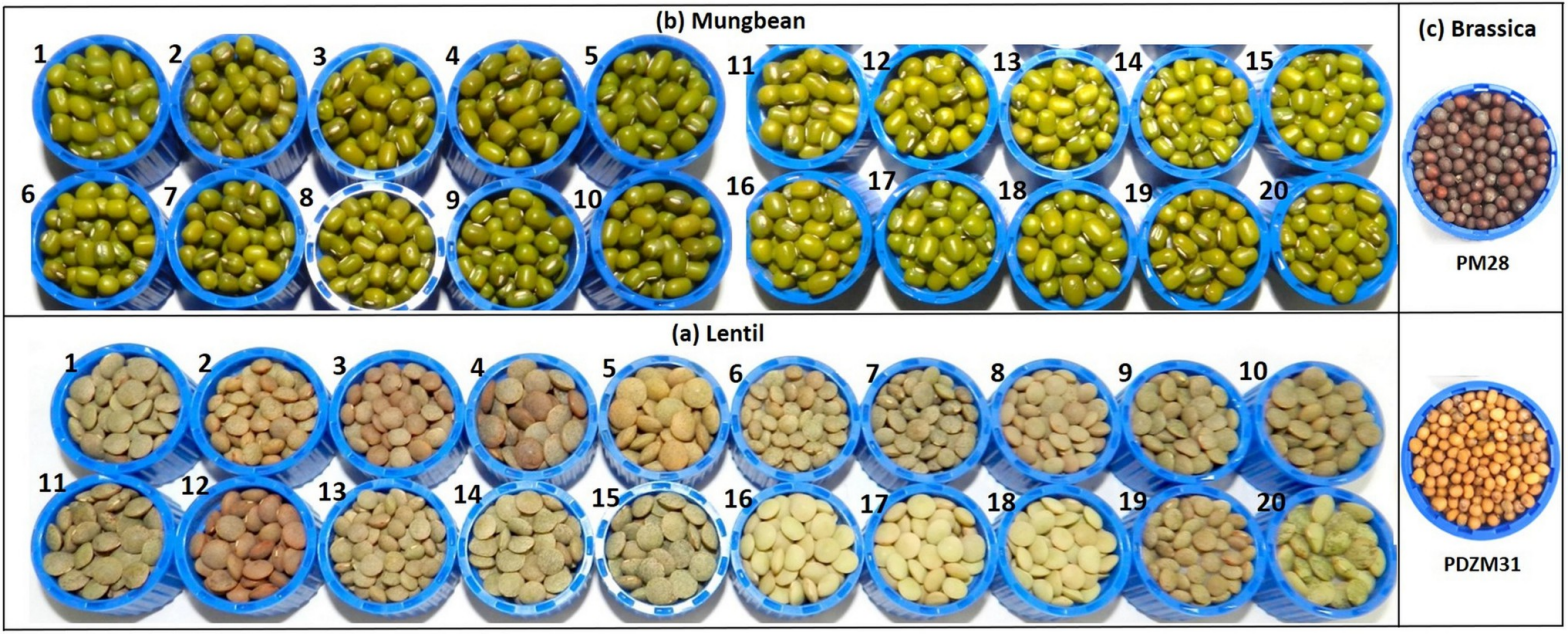
The mungbean, lentil, and Indian mustard genotypes used in the study. Where **(a)** mungbean genotypes are 1. Pusa Baisakhi, 2. Pusa Ratna, 3. Pusa Vishal, 4. Pusa105, 5. Pusa0672, 6. Pusa9072, 7. Pusa9531, 8. MH96-1, 9. MH318, 10. MH421, 11. MH521, 12. MH810, 13. ML512, 14. ML818, 15. PS16, 16. TM 96–2, 17. IPM02-3, 18. IPM02-14, 19. IPM409-4, 20. PMR1; **(b)** lentil genotypes are 1. L4076, 2. L4147, 3. L4594, 4. L7903, 5. HM1, 6. BM4, 7. JL1, 8. Sehore74-3, 9. NDL1, 10. IPL81, 11. IPL321, 12. K75, 13. KLS218, 14. DPL58, 15. DPL62, 16. PL1, 17. PL2, 18. PL6, 19. L830, 20. L4602; while **(c)** Indian mustard genotypes were 1. PM28 and 2. PDZM31.

To optimize the seeding density, harvesting stage, and marketable yield, seeds of 20 mungbean and lentil genotypes each and two Indian mustard genotypes (Table 1; Fig 1) were sown in three replications with different seeding-density: two-seed/cm^2^, three-seeds/cm^2^, and four-seeds/cm^2^ for mungbean and lentil, while six, eight, and ten seeds/cm^2^ for Indian mustard. The selected mungbean and lentil genotypes were very diverse for several parameters including antioxidant activities and the mineral profiles [3]. In addition, PDZM31 (Pusa Double Zero Mustard-31) is the first double zero (erucic acid <2% and glucosinolates <30ppm) mustard variety of India [32], while PM28 is a short duration variety. Microgreens are commercially produced under partially controlled conditions on different growing-medium like cocopeat or a combination of cocopeat, vermiculite, and sand. In addition, to avoid any variations (in quality of the produce) due to the growing conditions (temperature, photoperiod, etc.), the genotypes were grown under partially controlled conditions in the National Phytotron Facility, IARI, New Delhi which is located at the latitude, longitude, and altitude of 28.6412° N, 77.1627° E, and 228.61 m AMSL, respectively. The desired temperature was maintained for mungbean (28/26°C), lentils (21/18°C), and Indian mustard (21/18°C) along with a 10:14 h of day and night cycles.

**Table 1:** Genotypes, seed-weight, and moisture content in the studied lentil, mungbean, and Indian mustard microgreens.

**S. No**.	**(a) Mungbean microgreens**			**(b) Lentil microgreens**		
	**Genotype**	**10 seed weight (g)**	**Moisture (%)**	**Genotype**	**10 seed Weight (g)**	**Moisture (%)**
1	Pusa Baisakhi	.37±.02	92.76±1.03	L4076	.21±.006	83.59±1.64
2	Pusa Ratna	.41±.01	91.14±.69	L4147	.25±.015	85.53±1.73
3	Pusa Vishal	.54±.015	90.01±1.44	L4594	.35±.01	83.99±2.27
4	Pusa105	.43±.021	90.46±.82	L7903	.35±.02	83.59±2.24
5	Pusa0672	.42±.02	92.59±2.00	HM1	.37±.06	85.70±1.52
6	Pusa9072	.40±.031	90.13±.93	BM4	.22±.03	84.21±2.35
7	Pusa9531	.33±.015	91.77±1.32	JL1	.27±.015	84.55±1.37
8	MH96-1	.40±.021	92.00±1.41	Sehore74-3	.24±.02	84.32±1.81
9	MH318	.48±.01	92.08±1.35	NDL1	.22±.06	85.21±1.13
10	MH421	.40±.017	92.46±1.66	IPL81	.21±.06	85.62±1.11
11	MH521	.38±.06	92.22±.86	IPL321	.34±.01	83.97±1.90
12	MH810	.33±.06	92.41±2.30	K75	.28±.015	82.69±2.48
13	ML512	.31±.015	92.78±.92	KLS218	.21±.006	85.19±1.28
14	ML818	.37±.08	92.92±1.43	DPL58	.32±.015	84.61±2.45
15	PS16	.30±.01	91.70±1.01	DPL62	.33±.006	84.37±1.76
16	TM96-2	.36±.02	91.76±1.33	PL1	.38±.015	83.45±1.28
17	IPM02-3	.41±.012	93.06±1.07	PL2	.30±.03	83.65±1.96
18	IPM02-14	.41±.08	91.95±1.34	PL6	.28±.015	84.95±1.39
19	IPM409-4	.32±.06	91.54±1.78	L830	.20±.006	85.95±1.01
20	PMR-1	.35±.012	91.80±1.02	L4602	.39±.01	82.93±1.29
**(c) Indian mustard microgreens (100 seed weight, g)**						
1.	PM-28	.44±0.03	90.5±1.3	2. PDZM-31	.33±0.02	89.83±1.9

Where values are expressed as mean±SD (n = 3).

Freshly harvested seeds (Table 1) were obtained from the Division of Genetics, IARI, New Delhi having more than 90% germination. The seeds were surface-sterilized at room temperature (24°C) for one minute in 1% sodium hypochlorite (NaOCI) solution and rinsed twice with sterile water [33] and then sown in plastic trays (38×28×6 cm) in three replicates. The autoclaved (120°C, 120 Pa, 90 min) growing-medium consisted of coco peat: vermiculite: sand (2:1:1) was used for growing these microgreens. Based on the growth rate, harvesting was performed at different durations for mungbean (after 5^th^, 7^th^, and 9^th^ day), lentil (after 7^th^, 9^th^, and 11^th^ day), and Indian mustard (after 6^th^, 8^th^ and 10^th^ day) microgreens.

Microgreens were harvested using ethanol-cleaned scissors by cutting the stem approximately 1.0 cm above the growing medium and were immediately weighed using analytical balance to determine the total fresh weight (FW). Afterward, these were dried in hot air GenLab vertical oven (40°C for 72h), then weighted and kept in an airtight container for further biochemical analysis. The moisture content was calculated as per the equation:

$$Moisture\;\left(\%\right)=\left[\frac{\left\{Initial\;Weight\;\left(g\right)-Final\;weight\;(g)\right\}}{Initial\;Weight\;(g)}\;\times 100\right]$$

Where, Initial weight (g) = Weight of fresh microgreens after harvesting; Final weight (g) = Weight of microgreens after 72h of drying at 40°C

### Microgreens microbial load analysis

The incidence of total aerobic bacteria (TAB), yeast & mold (Y&M), *Salmonella* spp., *Shigella* spp., *Listeria* spp., and *E*. *coli* were assessed for both unwashed (freshly harvested) and washed samples of mungbean (genotypes MH-810, MH-318, PS-16), lentil (genotypes K75, L4594, L830), and Indian mustard (genotypes PM28, PDZM31) microgreens as obtained from the partially controlled conditions. The washing was performed using double distilled water for two minutes and then samples were air-dried in the laminar airflow (Svision, India). The mungbean, Indian mustard, and lentil microgreens were harvested on 7^th^, 8^th^, and 9^th^ day of sowing, respectively, and were stored in zip lock bags at 4°C and 1.0 g tissue was used to study the microbial load at 1^st^, 2^nd^, 4^th^, 8^th^, and 12^th^ day (day of harvest was considered 1^st^ day).

Microbial growth was assayed following the standard protocols [34, 35]. A 1.0 g sample was incubated in 10.0 mL sterile phosphate-buffered saline (PBS, 10x solution from Sigma Aldrich, USA) and vortexed (15 min). Plating of serially diluted samples (1.0 mL) was done on different agar plates. TAB population was identified by plating samples on nutrient agar (NA) supplemented with Amphotericin b (5.0mg/mL; an anti-fungal agent) and incubated at 37°C for 24–48h. Y&M enumeration was performed by plating samples on potato dextrose agar (PDA, Merck, Germany) supplemented with 50.0 mg/mL Chloramphenicol and incubated at 25°C for 48 to 72h.

*Salmonella* and *Shigella* were recorded (based on their colony morphology) by plating the samples on Xylose Lysine Deoxycholate agar (XLD, Merck, Germany) supplemented with Amphotericin b (5.0mg/mL) and incubated in dark at 35±2°C for 24 h. *Listeria* spp. was identified by plating the samples on Chromed Listeria Agar (Merck, Germany) supplemented with Nalidixic acid (13.0mg/mL), Ceftazidime (10.0mg/mL), and Amphotericin b (5.0mg/mL) and incubated at 37°C for 24 h. *E*. *coli* O157:H7 population was identified by plating the samples on Sorbitol MacConkey agar (Merck, Germany) and incubated at 37°C for 24 h. Each microbial count was determined as the mean of three measurements and the result was expressed as log CFU per g of tissue.

### Microgreens shelf life and sensory evaluation

For shelf life and sensory evaluation, two genotypes each of mungbean (MH810 and MH318), lentil (L830 and K75), and Indian mustard (PM28 and PDZM31) microgreens were harvested at the optimum stage and stored in food-grade linear low-density polyethylene (LLDPE) bags to avoid cross-contamination [30]. For mungbean and lentils, the genotypes having largest and smallest seed sizes were selected. The LLDPE bags are of 16×12 cm size (8.0 g per bag) and 51μ thickness. The samples were then stored in three replications at 4°C (in dark) for different durations. A panel of seven semi-trained judges (aged 24–45 years) from the IARI, New Delhi (India) performed the sensory evaluation [36]. Sensory evaluation (color & appearance, aroma, taste, and overall acceptability) was performed after 2^nd^, 4^th^, and 6^th^ day of storage using a 10-point hedonic scale (10 = like ultimate, 9 = like extremely, 8 = like strongly, 7 = like moderately, 6 = like slightly, 5 = neither like nor dislike, 4 = dislike slightly; 3 = dislike moderately, 2 = dislike strongly, and 1 = dislike extremely [34]. A score of 6 was considered as the limit of salability [37]. A sample size of 2.0 g of each microgreen was used for evaluation.

### Electrolyte leakage analysis

The electrolyte leakage of freshly harvested and stored microgreens was measured to find the possible tissue deterioration during storage. For this, 20.0g microgreens sample (from each replicate) was dipped in 400 mL deionized double distilled water (at 20 °C) and gently shaken for 30.0 min. The solution conductivity (μs/cm) was then measured using a conductivity meter (Orion 4-star portable pH/conductivity meter; Thermo Electron Corporation, U.S.A.) by dipping the probe in the sample solution [29, 38].

### Statistical analysis

The experiments were conducted thrice and the results were presented as mean±SD. One-way analysis of variance (ANOVA) was performed using SPSS11.5 to compare the groups, and Pearson’s correlation test was used to assess the correlation between means. The mean comparison was performed using Tukey’s test and a *P*≤0.05 was regarded as significant.

## Results and discussion

There has been a growing interest in promoting a healthy lifestyle including consumption of nutritious and quality foods, and microgreens offer a very good option [27, 38–40]. However, growing conditions of microgreens affect their overall yield, while their storage for some time may facilitate the growth of certain harmful microorganisms having food safety risks. Thus, this study was aimed to find the optimum growth parameters, sensory details, and existing microbial load during storage of mungbean, lentil, and Indian mustard microgreens.

### Optimization of microgreens yield

The growing medium is very crucial for proper germination, growth of microgreens and its physical properties including porosity and water holding capacity [41]. Three combinations of growing medium consisting of cocopeat: vermiculite: sand in the ratio of 1:1:1, 2:1:1, and 2:2:1 was studied and 2:1:1 medium was found best in terms of water holding capacity. For this growing-medium combination, we generally need only 1–2 light irrigation when microgreens were grown in plastic trays (without holes) under greenhouse conditions [42]. The trays were placed on a leveled surface on benches [24] and two most common factors affecting the total yield include seeding density and plant growth. Also, harvesting microgreens at the right stage is the key production strategy, since the time from sowing to harvesting varies greatly from crop to crop [3, 42].

**Fig 2:**
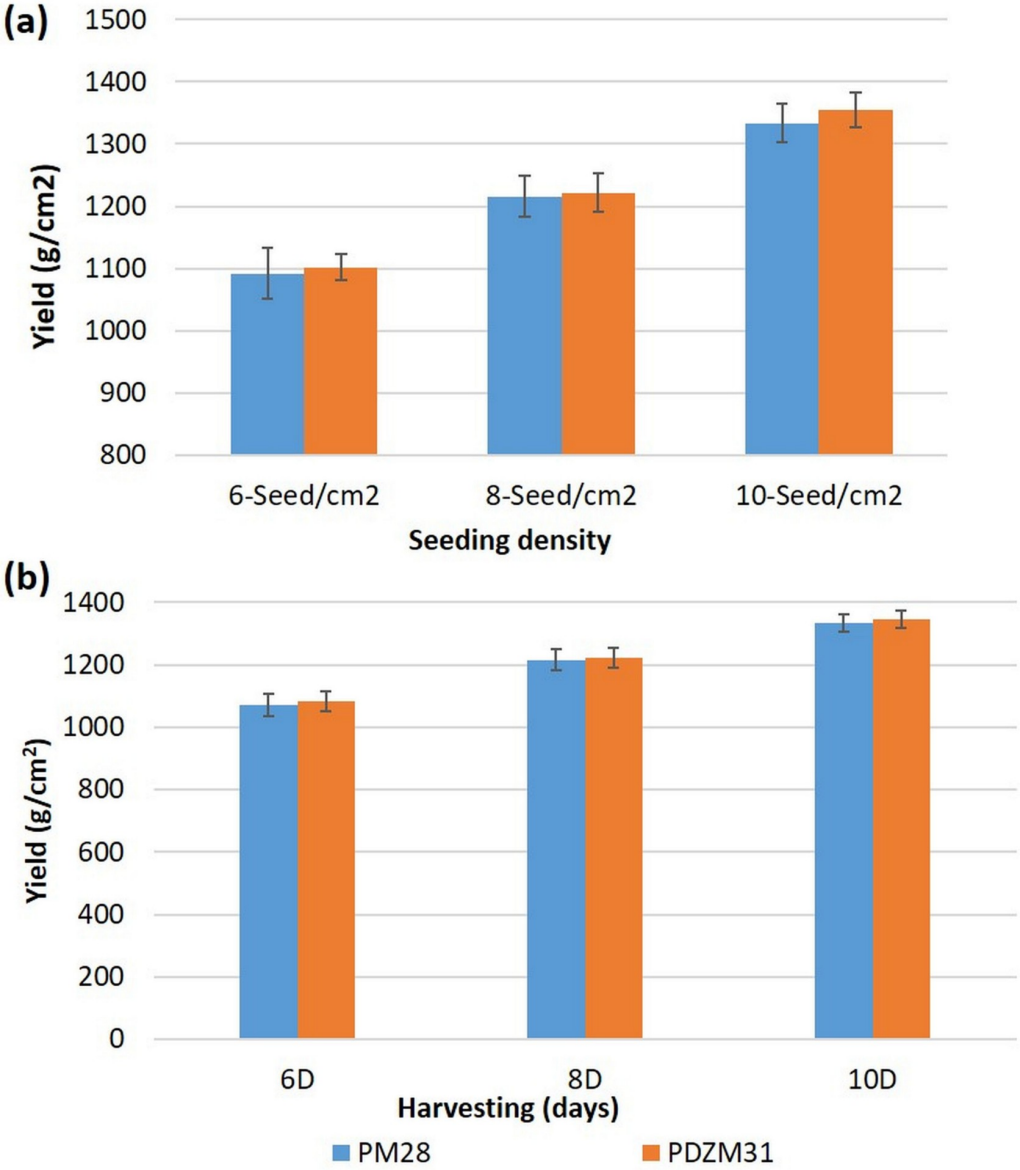
Microgreens yield of two Indian mustard genotypes (PM28 & PDZM31) (a) at a seeding density of 6, 8, and 10 seed/cm^2^ on 8^th^ day after sowing and (b) at 6^th^, 8^th^, and 10^th^ day of sowing. Where ‘D’ is days after sowing and values are expressed as mean±SD (n = 3).

Optimum seeding density is very specific to the crop species and is generally based on mean seed weight and germination (%) [42]. The yield of microgreens in this study showed an increasing trend with increasing seeding-density (Table 2a and 2b, Fig 2a). But, once it crossed the optimum seeding density, the marketable quality of microgreens got deteriorated. In mungbean, the microgreens yield at 2-seed/cm^2^ was recorded from 1854.38±19.69 to 2168.47±55.70 g/m^2^, while at 4-seed/cm^2^ this was 2123.35±17.74 to 2572.00±47.76 g/m^2^. Similarly, in lentils, the microgreens yield recorded at 2-seed/cm^2^ was 899.45±8.41 to 1222.56±60.79 g/m^2^ while at 4-seed/cm^2^ this was 1129.10±29.31 to 1417.90±56.23 g/m^2^. The yield of Indian mustard microgreens at 6-seed/cm^2^ ranged from 1091.56±41.09 to 1101.70±21.37 g/m^2^, while at 10 seed/cm^2^ this was from 1333.88±31.32 to 1355.78±28.04 g/m^2^. The yield details are presented in Table 2a and 2b and Fig 2a. For mungbean, and lentils 3-seed/cm^2^ was found optimum, while for Indian mustard it was 8-seed/cm^2^. Any increase in the seeding density beyond optimum resulted in poor marketable-quality produce. Excessive plant stand resulted in undesirably elongated shoots (due to more congestion and competition). Higher seeding density also hampered air circulation, favorable for fungal growth [43]. In addition, increase in seeding density result in higher seed cost.

**Table 2:** Lentil and mungbean microgreens yield of twenty genotypes each at different seeding densities.

S. No.	Genotype (a) Mungbean	Yield (g/m^2^)			Genotype (b) Lentil	Yield (g/m^2^)		
		2-Seed/cm^2^	3-Seed/cm^2^	4-Seed/cm^2^		2-Seed/cm^2^	3-Seed/cm^2^	4-Seed/cm^2^
1	**Pusa Baisakhi**	***1854*.*38±19*.*69e***	***1952*.*94±36*.*63g***	***2125*.*46±18*.*68f***	L4076	948.60±36.28defg	1059.79±64.44de	1192.44±40.53c
2	Pusa Ratna	2080.59±49.80abc	2258.32±54.82bcd	2359.00±54.75bcd	L4147	976.54±47.54defg	1083.73±86.59bcde	1186.88±81.20c
3	**Pusa Vishal**	***2168*.*47±55*.*70a***	***2450*.*11±46*.*45a***	***2572*.*00±47*.*76a***	L4594	1068.92±77.35abcdef	1206.95±82.17abcd	1308.94±66.62abc
4	Pusa105	2137.43±60.88ab	2348.23±73.03abc	2449.06±54.31abc	L7903	1073.90±29.56abcdef	1165.06±62.68abcde	1293.067±87.81abc
5	Pusa0672	2010.75±21.22abcde	2170.39±51.06cdef	2306.78±64.19cde	HM1	1093.66±75.70abcde	1146.97±84.08abcde	1270.763±85.09abc
6	Pusa9072	2143.69±50.95a	2396.13±50.25ab	2498.30±61.15ab	BM4	962.85±36.99defg	1071.18±77.97cde	1219.927±48.80bc
7	Pusa9531	1922.23±59.60de	2021.69±66.35fg	2215.75±70.09def	JL1	900.63±27.88g	1053.69±98.26de	1213.727±94.52bc
8	MH96-1	1886.18±94.93e	1974.84±48.69g	2159.79±71.78ef	Sehore74-3	941.36±45.25efg	1024.47±28.27de	1129.1±29.31c
9	MH318	2078.95±41.11abcd	2359.50±81.37abc	2461.43±69.90abc	NDL-1	899.45±8.41g	1028.14±58.64de	1148.75±79.44c
10	MH421	1946.48±66.58cde	2069.36±83.16defg	2213.84±47.42def	IPL81	934.37±66.02fg	1061.88±69.52de	1215.28±58.52bc
11	MH521	1953.81±25.14cde	2063.26±33.97efg	2200.01±40.77def	IPL321	1147.73±55.78abc	1281.78±29.61ab	1393.13±84.96ab
12	MH810	1961.55±51.24cde	2128.19±54.61defg	2222.92±66.69def	K75	1009.39±19.00cdefg	1108.51±80.06abcde	1241.32±54.64abc
13	ML512	1983.22±16.43bcde	2116.78±68.51defg	2237.32±60.56def	KLS218	1060.88±67.23bcdef	1127.87±92.47abcde	1292.173±37.23abc
14	ML818	1950.20±52.34cde	2088.94±85.37defg	2193.02±82.51def	DPL58	1105.28±41.91abcd	1190.85±60.31abcd	1300.613±13.18abc
15	PS16	1906.26±12.39e	1986.40±49.02fg	2123.35±17.74f	**DPL62**	***1222*.*56±60*.*79a***	***1283*.*51±36*.*74ab***	***1417*.*9±56*.*23a***
16	TM96-2	1918.21±22.95e	2045.65±63.86efg	2241.09±55.77def	PL1	1176.23±41.11ab	1272.97±67.25abc	1404.6±57.61ab
17	IPM02-3	1980.96±43.88bcde	2173.12±81.03cdef	2307.45±38.02cde	PL2	1078.94±84.25abcdef	1201.25±48.27abcd	1322.463±47.53abc
18	IPM02-14	1978.93±37.96cde	2213.03±67.78bcde	2348.99±46.91bcd	PL6	977.05±6.30defg	1044.06±48.41de	1171.65±49.79c
19	IPM409-4	1939.30±93.27cde	2110.64±66.59defg	2219.83±50.50def	**L830**	***900*.*80±28*.*73g***	***977*.*14±52*.*80e***	***1138*.*75±57*.*31c***
20	PMR-1	1891.20±42.48e	1996.22±21.63fg	2161.29±70.50ef	L4602	1171.95±65.19ab	1289.38±31.36a	1392.777±36.13ab

Where mungbean at 07^th^ day, while lentil was harvested on 09^th^ day after sowing. Values are expressed as mean±SD (n = 3) and different letters indicate a significant difference (*P*≤.05). Values in bold represent maximum and minimum values.

Day of harvesting is also equally important for reaping the best marketable-quality yield. On 5^th^ to 9^th^ day, the mungbean microgreens yield ranged from 1773.87±30.32 to 2645.06±52.60 g/m^2^; while on 7^th^ to 11^th^ day, the lentil microgreens yield ranged from 840.14±45.87 to 1411.29±77.66 g/m^2^ (Table 2) and the Indian mustard microgreens yield on 6^th^ to 10^th^ day ranged from 1070.66±35.13 to 1346.87±27.27 g/m^2^ (Fig 2b).

Different microgreens species have different harvesting stages to achieve their marketable hypocotyl length and leaf area to reap maximum economic benefit. The mungbean, Indian mustard, and lentil microgreens have different growth rates and under studied conditions; 7^th^, 8^th^, and 9^th^ day, respectively were found optimum for harvesting (Table 3; Fig 2b). Even though the overall yield recorded was higher during the later stages of harvesting, the quality of microgreens got deteriorated (S1 Fig). Additionally, significant genotypic differences for yield were observed in the studied microgreens. Similarly, the microgreens yield was recorded as 659 g/m^2^ in *Brassica oleracea* L. and 1548 g/m^2^ in *Cichorium intybus* L. [27]. Also, the optimum days to harvest for radish microgreens were 7^th^ day, arugula–9^th^ day, and red cabbage–11^th^ day under specific growing conditions [28].

**Table 3:** Microgreens yield (g/m^2^) of twenty lentil and mungbean genotypes at different days of harvesting.

S. No.	(a) Genotype (Mungbean)	Yield (g/m^2^)			(b) Genotype (Lentil)	Yield (g/m^2^)		
		7^th^ Day	9^th^ Day	11^th^ Day		5^th^ Day	7^th^ Day	9^th^ Day
1	**Pusa Baisakhi**	***1813*.*20±69*.*61ef***	***1952*.*94±36*.*63g***	***2110*.*05±68*.*40h***	L4076	981.79±47.04bcde	1059.79±64.44de	1163.15±57.80cde
2	Pusa Ratna	2102.33±80.50abc	2258.32±54.82bcd	2386.95±47.67bcde	L4147	994.15±41.39bcde	1083.73±86.59bcde	1122.82±78.66e
3	**Pusa Vishal**	***2160*.*84±51*.*31a***	***2450*.*11±46*.*45a***	***2557*.*94±67*.*71ab***	L4594	1070.23±55.60abcd	1206.95±82.17abcd	1248.57±51.35abcde
4	Pusa105	2057.28±53.13abcd	2348.23±73.03abc	2645.06±52.60a	L7903	1047.04±52.06bcd	1165.06±62.68abcde	1209.58±33.43bcde
5	Pusa0672	1908.95±51.06def	2170.39±51.06cdef	2372.25±55.92cde	HM1	1015.87±47.39bcd	1146.97±84.08abcde	1209.25±26.04bcde
6	Pusa9072	2111.14±59.04ab	2396.13±50.25ab	2478.54±90.27abcd	BM4	990.60±78.03bcde	1071.18±77.97cde	1246.52±55.82abcde
7	Pusa9531	1887.31±60.65def	2021.69±66.35fg	2135.67±51.02gh	JL1	1005.93±49.32bcd	1053.69±98.26de	1159.83±58.29de
8	MH96-1	1871.41±49.61ef	1974.84±48.69g	2154.17±46.56fgh	Sehore74-3	982.52±28.62bcde	1024.47±28.27de	1238.19±31.39bcde
9	MH318	2053.67±77.74abcd	2359.50±81.37abc	2543.63±59.45abc	NDL-1	992.53±33.90bcd	1028.14±58.64de	1164.14±53.29cde
10	MH421	1864.41±66.45ef	2069.36±83.16defg	2143.01±59.81gh	IPL81	957.97±59.78de	1061.88±69.52de	1177.89±84.45bcde
11	MH521	1973.14±81.49bcde	2063.26±33.97efg	2243.03±62.12efgh	IPL321	1125.73±54.69abc	1281.78±29.61ab	1331.90±45.54ab
12	MH810	1894.74±34.21def	2128.19±54.61defg	2152.58±55.69fgh	K75	1062.66±56.23bcd	1108.51±80.06abcde	1218.14±51.26bcde
13	ML512	1930.35±50.62cdef	2116.78±68.51defg	2283.53±67.24efgh	KLS218	974.57±41.62cde	1127.87±92.47abcde	1221.73±45.03bcde
14	ML818	1901.11±46.00def	2088.94±85.37defg	2304.72±36.49defg	DPL58	1011.25±30.24bcd	1190.85±60.31abcd	1224.81±45.74bcde
15	PS16	1778.65±28.04f	1986.40±49.02fg	2176.42±51.58fgh	**DPL62**	***1214*.*01±47*.*72a***	**1283.51±36.74ab**	***1411*.*29±77*.*66a***
16	TM96-2	1849.26±52.26ef	2045.65±63.86efg	2136.16±39.63gh	PL1	1055.90±57.94bcd	1272.97±67.25abc	1315.32±62.96abcd
17	IPM02-3	1932.44±49.40cdef	2173.12±81.03cdef	2275.52±88.07efgh	PL2	990.95±33.18bcde	1201.25±48.27abcd	1305.84±29.26abcd
18	IPM02-14	1955.46±48.08bcde	2213.03±67.78bcde	2323.77±38.86def	PL6	975.74±55.82cde	1044.06±48.41de	1228.81±39.22bcde
19	IPM409-4	1869.92±55.11ef	2110.64±66.59defg	2279.31±41.62efgh	**L830**	***840*.*14±45*.*87e***	***977*.*14±52*.*80e***	***1118*.*75±34*.*04e***
20	PMR-1	1773.87±30.32f	1996.22±21.63fg	2120.55±32.77h	L4602	1129.84±32.26ab	***1289*.*38±31*.*36a***	1328.77±61.09abc

Where values are expressed as mean±SD (n = 3) and different letters indicate a significant difference (*P*≤.05). Values in bold represent maximum and minimum values.

A very high correlation was recorded between mean seed weight and yield in both mungbean (r^2^ = .73) and lentil (r^2^ = .78) genotypes. As we used only two Indian mustard samples with nearly the same seed weight, the correlation analysis could not be performed.

### Microbial counts

In many countries, various microbial outbreaks have been reported mainly due to the consumption of contaminated sprouts [19–21]. Thus, it becomes imperative to monitor and evaluate the microbial load in the microgreens too. Among different studied microbes, we have recorded the growth of Y&M, TAB, *Shigella*, and *E*. *coli* (O157:H7) in the studied microgreens. The target pathogenic bacteria *Salmonella* spp. and *Listeria* spp. were not detected in any of the tested samples. On contrary, Bergšpica et al. [11] have detected the presence of *Listeria innocua* in the radish and sunflower microgreens, and *Salmonella* spp. in sunflower microgreens. In general, *Listeria innocua* is considered to be a non-pathogenic *Listeria* species [44].

Washing of microgreens was done using double distilled water for 2-minutes and results of both washed and unwashed samples for the growth of various microbes in mungbean was found comparable to lentil and mustard microgreens (Table 4). On contrary, Chandra et al. [29] reported a very high value for the TAB count (7.8 logCFU/g) of unwashed cabbage microgreens; which after washing get reduced to 7.2 logCFU/g. Washing has shown a significant (*P*≤0.05) reduction in the TAB (2.6 to 3.4 logCFU/g) and Y&M (1.1 to 2.2 logCFU/g) over fresh-cut beetroot samples [45]. Survival of *E*. *coli* O157:H7 was reported on radish [46], arugula, kale, lettuce, and mizuna microgreens [47]; while Di Gioia et al. [48] reported microbial growth on brassica microgreens. Inoculation of seed and irrigation water with Shiga toxin-producing *E*. *coli* (STEC) has resulted in the growth of bacteria on eight microgreens species [49].

**Table 4:** Microbial load in three genotypes each of lentil (K75, L4594 & L830) and mungbean (MH810, MH318 & PS16) and two genotypes of Indian mustard (PM28, PDZM-31) microgreens after 1^st^, 2^nd^, 4^th^, 8^th^, and 12^th^ day of storage at 4°C under washed and unwashed conditions.

Microbes	Genotype	Day-1		Day-2		Day-4		Day-8		Day-12	
**(a)**	**Mungbean**	**Unwashed**	**Washed**	**Unwashed**	**Washed**	**Unwashed**	**Washed**	**Unwashed**	**Washed**	**Unwashed**	**Washed**
**TAB**	MH810	3.320±.513bc	2.810±.179bc	3.590±.250bc	3.210±0.513b	3.653±0.340bc	3.041±.590bc	3.531±.490cd	2.699±.410abc	3.710±.480abc	2.870±.210ab
	MH318	3.980±.390ab	3.650±.767a	4.060±.280ab	3.820±0.419a	4.193±0.320ab	3.982±.170a	4.009±.290abc	3.120±.570a	4.000±.270abc	3.000±.610ab
	PS16	3.650±.550abc	3.320±.430ab	3.830±.146b	3.430±0.290ab	4.041±0.450abc	3.431±.260ab	3.875±.198bcd	3.000±.340ab	3.900±.430abc	3.560±.510a
**Y&M**	MH810	1.560±.290d	1.640±.386ef	1.980±.185e	1.880±0.430de	2.146±0.120d	1.954±.250de	1.699±.660f	1.301±.240f	1.900±.240f	1.600±.330d
	MH318	2.050±.120d	1.340±.423f	2.140±.290de	2.100±0.443d	2.230±0.490d	2.000±.350de	2.477±.190e	2.380±.410cd	2.580±.740e	2.500±.690bc
	PS16	1.950±.120d	1.570±.262ef	2.060±.540de	2.230±0.160d	2.146±0.240d	2.114±.160de	2.380±.300e	1.954±.340de	2.560±.440ef	2.000±.320cd
***E*. *coli***	MH810	2.180±.625d	2.140±.250de	2.520±.141d	2.350±0.104cd	2.699±0.640d	2.477±.140cd	2.602±.420e	2.000±.510de	2.800±.330de	2.200±.690bcd
	MH318	3.480±.443abc	3.060±.179ab	4.394±.290a	3.410±0.328ab	4.013±0.560abc	3.556±.020ab	4.590±.540a	3.255±.054a	4.340±.490a	3.450±.070a
	PS16	3.450±.410abc	2.420±.513cd	3.610±.290bc	2.930±0.350bc	3.973±0.150abc	3.176±.480b	3.568±.236cd	2.477±.125bcd	3.630±.017bc	2.980±.240ab
***Shigella***	MH810	3.07±.40c	1.020±.040f	3.14±.560c	1.230±0.230f	3.40±0.25c	1.210±.310f	3.30±.65d	1.350±.150f	3.40±.41cd	1.540±.680d
	MH318	3.97±.61ab	1.040±.280f	4.06±.190ab	1.150±0.419f	4.35±0.39a	1.200±.540f	4.51±.03ab	1.580±.240ef	4.11±.14ab	1.620±.570d
	PS16	4.12±.65a	1.190±.174f	4.37±.240a	1.3800.328ef	4.54±0.25a	1.640±.510ef	4.09±.23abc	2.477±.120bcd	4.21±.31ab	2.300±.260bcd
**(b)**	**Lentil**	**Unwashed**	**Washed**	**Unwashed**	**Washed**	**Unwashed**	**Washed**	**Unwashed**	**Washed**	**Unwashed**	**Washed**
**TAB**	K75	4.594±.015a	4.491±.030a	4.963±.038a	4.792±.014a	4.990±.020a	4.778±.031a	5.147±.107b	4.881±.024a	5.327±.047ab	4.979±.023a
	L4594	4.803±.121a	4.602±.048a	4.970±.026a	4.771±.038a	5.093±.068a	4.778±.056a	5.259±.053ab	4.903±.011a	5.390±.076a	5.000±.139a
	L830	4.772±.100a	4.491±.049a	4.970±.032a	4.841±.014a	5.218±.174a	4.949±.054a	5.364±.064a	4.881±.017a	5.406±.241a	4.976±.023a
**Y&M**	K75	4.058±.155b	3.771±.030b	4.047±.133b	3.845±.021b	4.256±.116b	3.944±.017b	4.524±.090c	3.954±.015b	5.119±.097abc	4.869±.039ab
	L4594	4.109±.120b	3.794±.017b	4.211±.013b	3.847±.009b	4.342±.045b	3.936±.013b	4.663±.076c	4.040±.040b	4.983±.103c	4.597±.050c
	L830	4.021±.102b	3.785±.015b	4.161±.159b	3.833±.010b	4.335±.073b	3.914±.008b	4.512±.033c	3.968±.012b	5.043±.060bc	4.716±.060bc
***E*. *coli***	K75	3.160±.161c	1.893±.025cd	3.239±.232cd	2.006±.092cd	3.367±.240c	2.335±.254cd	3.492±.272d	2.510±.166cd	3.766±.368ef	2.767±.169e
	L4594	3.037±.152c	1.903±.071cd	3.237±.356cd	2.087±.185c	3.337±.412c	2.530±.321c	3.540±.062d	2.797±.190c	4.247±.234d	3.064±.136d
	L830	3.170±.219c	1.890±.027cd	3.273±.060c	1.994±.016cd	3.457±.077c	2.179±.206d	3.553±.101d	2.430±.504d	3.987±.110de	2.880±.358de
***Shigella***	K75	2.813±.117d	1.900±.139cd	3.023±.067d	1.990±.020cd	3.223±.050c	2.083±.025d	3.417±.110d	2.106±.077e	3.673±.068f	2.236±.116f
	L4594	2.984±.055cd	1.760±.235d	3.148±.054cd	1.967±.045d	3.227±.081c	2.170±.200d	3.494±.014d	2.297±.097de	3.712±.172ef	2.313±.088f
	L830	3.070±.115c	1.927±.030c	3.187±.021cd	1.960±.036d	3.457±.148c	2.187±.211d	3.580±.173d	2.263±.264de	3.690±.250ef	2.350±.157f
**(c)**	**Indian mustard**	**Unwashed**	**Washed**	**Unwashed**	**Washed**	**Unwashed**	**Washed**	**Unwashed**	**Washed**	**Unwashed**	**Washed**
**TAB**	PM28	4.39±.13a	3.67±.76ab	4.71±.44a	4.59±.26a	4.82±.29a	4.60±.40ab	5.19±.65a	4.54±.37a	5.26±.19a	4.65±.15ab
	PDZM31	4.14±.39a	3.84±.77ab	4.68±.51a	4.60±.42a	4.84±.33a	4.25±.18ab	5.17±.67a	4.28±.64a	5.29±.18a	4.88±.28a
**Y&M**	PM28	2.49±.17c	1.71±.54c	2.20±.17c	2.6±.18c	2.32±.39d	2.20±.42c	2.41±.14b	2.22±.22c	2.35±.07d	2.17±.09e
	PDZM31	1.37±.11d	1.59±.33c	2.10±.14c	2.17±.15c	2.37±.10d	2.17±.24c	2.39±.12b	2.08±.31c	2.31±.02d	2.20±.08e
***E*. *coli***	PM28	4.57±.24a	4.00±.36ab	4.90±.65a	4.53±.28a	4.49±.29ab	4.94±.04a	4.82±.62a	4.31±.14a	4.73±.28bc	4.07±.10cd
	PDZM31	4.54±.51a	4.42±.45a	4.70±.17a	4.52±.12a	4.88±.23a	4.08±.61b	4.59±.38a	4.18±.28ab	4.97±.70abc	4.39±.15bc
***Shigella***	PM28	3.40±.54b	3.20±.23b	3.60±.25b	3.60±.12b	3.90±.16c	3.90±.46b	4.28±.87a	4.20±.17ab	4.49±.10c	3.89±.11d
	PDZM31	4.30±.14a	3.90±.29ab	4.70±.55a	3.60±.43b	4.30±.19bc	3.90±.56b	4.74±.78a	3.63±.30b	5.13±.23ab	4.53±.47ab

Where Y&M: Yeast & mold, TAB: total aerobic bacteria. All the microbial counts are expressed in logCFU/g of microgreens. Values are expressed as mean±SD (n = 3) and different letters indicate a significant difference (*P*≤.05). Values in bold represent maximum and minimum values

No significant difference was recorded in the overall microbial load between the microgreens of different studied genotypes (Table 4a–4c). In general, an increasing trend was recorded for various microbial counts when samples were stored at 4°C from 1^st^ day to 12^th^ day (S2 Fig). Similarly, Chandra et al. [29] also recorded an increasing trend in the microbial population during storage of Chinese cabbage and beetroot samples [45].

In mungbean microgreens, washing significantly decreased the load of *Shigella* spp., whereas, amicrobial count (TAB, Y&M, and *E*. *coli*) did not show any significant decreasing effect (Table 4a). However, in lentil microgreens, washing significantly reduced the overall load of *Shigella* spp. and *E*. *coli*; but TAB and Y&M count did not show much reduction (Table 4b). This means that the survival and growth need of *Shigella* spp. and *E*. *coli* are different from that of aerobic bacteria and Y&M in the microgreens, as also recorded by Chandra et al. [45]. In mustard microgreens, although washing reduced overall microbial load, it was not very significant (Table 4c).

Washing of harvested microgreens has been practiced to remove the attached soil particles, to reduce the initial microbial load, and also for clean packaging. However, washing reportedly creates humid environmental conditions suitable for microbial growth, thus necessitating careful removal of excess moisture without causing any damage to the greens [18]. Relatively faster loss of shelf life was reported for the washed radish [31] and buckwheat microgreens over unwashed microgreens; which could be due to the damage caused during washing and dewatering, and also the presence of excess moisture in washed microgreens packages [30].

Microgreens are prone to bacterial internalization as the bacteria present in the seeds can become part of endophytic microflora [50]. Also, during germination, the bacteria present in the rhizosphere are attracted by the seed exudates and may enter through the germinating radicals or secondary roots [50]. Therefore, once contaminated, it is nearly impossible to eliminate the microbes from the living plant system. Thus, sanitization or washing of harvested microgreens may not be a very effective control strategy. In addition, microgreens being very delicate are quite prone to the damage caused by any such treatments [18]. A much lower value of microbial load in the present experiments could be due to the good agronomic practices used during the growth of the microgreens including the use of freshly harvested seeds, seed treatment, autoclaving of growing media, and use of alcohol cleaned scissors while harvesting the microgreens.

### Microgreens shelf life and sensory evaluation

Since microgreens are very tender, thus are extremely vulnerable to dehydration and quality deterioration. Therefore, to maintain the quality and shelf life of microgreens, proper refrigeration and packaging become extremely crucial [18]. At the time of harvesting, microgreens have a very high respiration rate [29] and can be stored comfortably for nearly a week time at <5°C [30, 31]. Immediately after the harvest, microgreens should be washed and cooled (1–5°C) [26] or this can be marketed in trays with growing-medium [24]. Thus, two genotypes each of mungbean (MH810 & MH318), lentil (L830 and K75), and Indian mustard (PM28 & PDZM31) were used for the shelf life and sensory evaluation. These were stored at 4°C for 6 days and analyzed at 1^st^, 2^nd^, 4^th^ and 6^th^ day of storage. The visual appearance of microgreens declined gradually as the storage time increased under cool (4°C) conditions (S1 Fig). Mungbean and Indian mustard microgreens showed nearly 4-day shelf life, while lentil microgreens could be used till 6^th^ day of their storage in 51μ thick LLDPE zip-lock bags (16×12 cm) at 4°C conditions. On contrary, based on visual parameters, the shelf life of arugula, radish, and red cabbage was recorded as 14, 21, and 14 days, respectively at 4°C; whereas, at 10°C this was 7, 14, and 7 days, respectively [28].

Till 4^th^ day of the storage at 4°C, all the studied sensory parameters such as color and appearance, aroma, taste, and overall acceptability of the studied microgreens showed the hedonic score of >6, which was considered as the limit of salability [37]. However, on the 6^th^ day of storage, a drastic reduction in all the sensory parameters of mungbean and mustard microgreens was recorded. Interestingly, lentil microgreens showed >6 hedonic scores for all the studied sensory parameters, even on 6^th^ day of its storage (Table 5a–5c). This could be due to relatively less moisture content in the lentil microgreens over mungbean or Indian mustard microgreens. In general, the moisture content in mungbean, lentil, and Indian mustard microgreens ranged from 90.01±1.44 to 93.06±1.07, 82.69±2.48 to 85.95±1.01, and 89.83% to 90.5%, respectively. An inverse relationship was found between the moisture content and the shelf-life (and sensory qualities) in the studied microgreens. Many reports underlined the importance of temperature in prolonging the overall post-harvest shelf life of various fresh-cut products including microgreens [51–53]. A slower respiration rate at low temperature can be directly correlated with the lower rate of cellular metabolism and cause a direct effect on visual microgreens quality and hence increased self-life [28].

**Table 5:** Sensory details including color & appearance, aroma, taste and overall acceptability of (a) mungbean (MH810 & MH318), (b) lentil (L830 & K75), and (c) Indian mustard (PM28 & PDZM31) microgreens.

Genotypes	Storage (days)	Sensory Characters			
		Color & appearance	Aroma	Taste	Overall acceptability
**(a) Mungbean**					
MH810	D1	9.80±.076a	9.50±.177a	9.34±.261a	9.47±.255a
	D4	7.64±.261b	7.79±.290b	7.19±.422b	7.30±.282b
	D6	4.93±.492c	3.73±.301c	4.19±.666c	3.91±.488c
MH318	D1	9.66±.090a	9.30±.200a	9.50±.283a	9.23±.225a
	D4	7.93±.183b	7.57±.353b	7.53±.353b	7.30±.203b
	D6	4.67±.480c	3.56±.424c	4.41±.615c	4.16±.450c
**(b) Lentil**					
K75	D1	9.36±.226ab	8.99±.188b	9.06±.184b	9.06±.159b
	D4	9.24±.184b	8.59±.259c	8.56±.261c	8.60±.278c
	D6	7.34±.447c	7.20±.374d	7.16±.430d	7.14±.358d
L830	D1	9.66±.325a	9.56±.282a	9.57±.291a	9.53±.446a
	D4	9.47±.361ab	9.31±.247ab	9.11±.318b	8.99±.181b
	D6	7.36±.410c	7.49±.376d	7.33±.437d	7.30±.239d
**(c) Mustard**					
PDZM31	D1	9.47±.237a	9.09±.155b	9.24±.342ab	9.26±.206ab
	D4	9.09±.146b	9.01±.181b	8.86±.333bc	8.90±.120bc
	D6	5.43±.342c	5.24±.232d	4.71±.290d	5.16±.118d
PM28	D1	9.70±.256a	9.40±.355a	9.61±.467a	9.46±.607a
	D4	9.03±.167b	9.14±.232ab	8.77±.212c	8.61±.352c
	D6	5.57±.373c	5.61±.318c	4.76±.362d	5.30±.185d

Where values are expressed as mean±SD (n = 7) and different letters indicate a significant difference (*P*≤.05).

### Electrical conductivity (EC) of washed and unwashed microgreens

**Fig 3:**
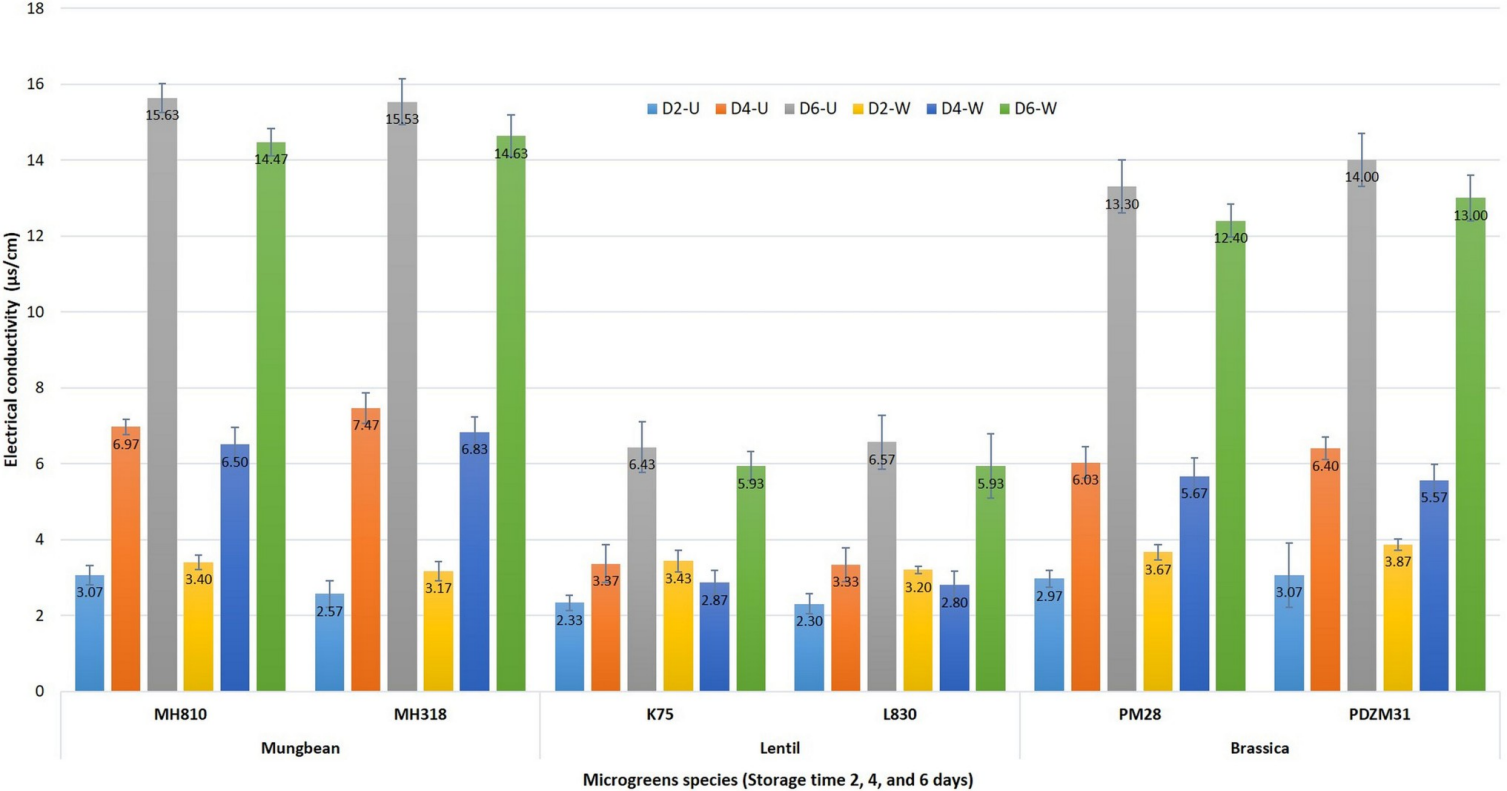
Changes in electrical conductivity of washed (W) and unwashed (U) microgreens samples (mungbean, lentil, and Indian mustard) when packed in LLDPE films during the 2^nd^, 4^th^, and 6^th^ day of storage at 4°C. Where D2-U, D4-U, and D6-U are ‘unwashed’; while D2-W, D4-W, and D6-W are ‘washed’ microgreens samples at 2^nd^, 4^th^, and 6^th^ day of storage, respectively. Values are expressed as mean±SD (n = 3) and different letters indicate a significant difference (*P*≤.05).

EC can be associated with the overall quality and shelf life of microgreens and is used as an indirect measure of the same [37]. With increasing storage duration, EC showed an increasing trend in both washed (with sterile double distilled water for 2.0 min) and unwashed microgreens over fresh samples (Fig 3). EC was recorded more for the mungbean microgreens, especially at 4^th^ day (6.97 μs/cm) and 6^th^ day (15.63 μs/cm) of storage over lentil (3.37 & 6.57 μs/cm) or Indian mustard (6.4 & 14.0 μs/cm) microgreens, respectively. Relatively less EC was recorded for 4^th^ and 6^th^-day samples under 4°C storage; while it was a bit more for washed samples for 2^nd^ day. Cell surface damage caused during washing treatment might have got repaired by 4^th^ day of storage. Interestingly, 6^th^ day of storage showed a sudden rise in EC for all the microgreens (Fig 3). Conductivity values showed a positive association with the storage duration of the studied microgreens.

On a similar note, the EC values showed a 7-fold increase (over initial value) for the tap water-washed Chinese cabbage microgreens, until the end of storage (9^th^ day), when packed in polypropylene (PP) film [29]. Similar observations were also recorded for fresh-cut cilantro [54]. An increase in EC values under storage may be due to the irreversible membrane damage and accumulation of CO_2_ from respiration [29]. On contrary, a decreasing trend in the electrolyte leakage was recorded for broccoli microgreens for various washing treatments and O3 washing (180s) under 09-day storage conditions [38].

## Conclusions

Rapid growth cycle, limited space requirement, rich flavor, diverse color, and highly economic produce makes microgreens a nutrient alternative that may contribute to the nutritional security of a large population. To the best of our knowledge, no study about the yield optimization and microbial aspects of mungbean, lentil, and Indian mustard microgreens has been reported so far from India. The use of good agricultural practice is the key to manage the microbial contamination of the growing microgreens [55]. More scientific information should be generated for various microgreens to eliminate the possibility of microbial contamination through seed, grow-media, grow-trays, and harvesting implements. In addition, post-harvest care such as harvesting at an optimum stage, proper sanitation, and maintenance of optimum temperature and humidity will help in longer storage and reduced risk of human pathogen contamination [12, 18, 56]. Thus, if grown and stored properly, there is no major risk of microbial illness from any kind of microgreens consumption. The success of microgreens technology will largely depend on the collective and collaborative efforts from the industry and researchers in the food-chemistry, biochemistry, genetics, and human nutrition working to enhance the yield and quality. This is the first such study from India, which included the microgreens of mungbean, lentil, and Indian mustard. Interestingly seeds of studied crops are readily available in any Indian kitchen, and we hope that the results will help in the popularization of these microgreens even at household levels.

## Supporting information

S1 Fig The difference in the growth pattern of mungbean microgreens (a) 4^th^ day of sowing and, (b) 9^th^ day after sowing (At a later stage the plants become lanky and of poor marketable quality). (TIF)

S2 Fig Representative figure showing microgreens of mungbean lentil, and Indian mustard stored for different durations at 4°C. (TIF)

## References

[1] Cohen MJ, Garrett JL (2010) The food price crisis and urban food (in) security. Environ Urbanization 22:467–482. doi: 10.1177/0956247810380375

[2] Sun J, Xiao Z, Lin LZ, Lester GE, Wang Q, Harnly JM, et al. (2013) Profiling polyphenols in five *Brassica* species microgreens by UHPLC-PDA-ESI/HRMS^n^. J Agril Food Chem 61:10960–10970. doi: 10.1021/jf401802n 24144328 PMC3915300

[3] Priti, Mishra GP, Dikshit HK, Vinutha T, Mechiya T, Stobdan T, et al. (2021) Diversity in phytochemical composition, antioxidant capacities, and nutrient contents among mungbean and lentil microgreens when grown at plain-altitude region (Delhi) and high-altitude region (Leh-Ladakh), India. Front in Plant Sciences, 12:710812. doi: 10.3389/fpls.2021.710812 34497624 PMC8420906

[4] Treadwell D, Hochmuth R, Landrum L, Laughlin W (2010) Microgreens: A new specialty crop. University of Florida, IFAS Extension. EDIS 2010(3). https://journals.flvc.org/edis/article/view/118552.

[5] Globe Newswire Report (2020) "United States Microgreens Market—Growth, Trends and Forecast (2020–2025)" https://www.reportlinker.com/p05916379/?utm_source=GNW. https://www.globenewswire.com/news-release/2020/07/02/2057048/0/en/United-States-Microgreens-Market-Growth-Trends-and-Forecast-2020-2025.html (Accessed on 30th June 2021)

[6] https://www.datamintelligence.com/research-report/microgreens-market

[7] www.researchandmarkets.com

[8] Puccinelli M, Malorgio F, Rosellini I, Pezzarossa B (2019) Production of selenium-biofortified microgreens from selenium-enriched seeds of basil. J Sci Food Agric 99:5601–5605. doi: 10.1002/jsfa.9826 31149731

[9] Di Gioia F, Petropoulos SA, Ozores-Hampton M, Morgan K, Rosskopf EN. Zinc and Iron Agronomic Biofortification of Brassicaceae Microgreens. *Agronomy*. 2019; 9(11):677. doi: 10.3390/agronomy9110677

[10] Ebert AW, Chang CH, Yan MR et al (2017) Nutritional composition of mungbean and soybean sprouts compared to their adult growth stage. Food Chem 237:15–22. doi: 10.1016/j.foodchem.2017.05.073 28763980

[11] Bergšpica I, Ozola A, Miltinxa E, Alksne L, Meistere I, Cibrovska A, et al. (2020) Occurrence of pathogenic and potentially pathogenic bacteria in microgreens, sprouts, and sprouted seeds on retail market in Riga, Latvia. Foodborne Pathogens and Disease 17(6):1–9. doi: 10.1089/fpd.2019.2733 31895586

[12] Riggio GM, Wang Q, Kniel KE, Gibson KE (2019) Microgreens—A review of food safety considerations along the farm to fork continuum. Int J Food Microbiol 290:76–85. doi: 10.1016/j.ijfoodmicro.2018.09.027 30308448

[13] CFIA-Canadian Food Inspection Agency (2018a) Lufa Farms Inc. brand Arugula Microgreens recalled due to *Salmonella*. Recalls and Safety Alerts. http://healthycanadians.gc.ca/recall-alert-rappel-avis/inspection/2018/67156r-eng.php

[14] Clark B (2017) *Salmonella* test prompts microgreens recall. Food Poison Journal. https://www.foodpoisonjournal.com/food-recall/salmonella-test-prompts-microgreens-recall/

[15] CFIA- Canadian Food Inspection Agency (2018b) Food recall warning—Goodleaf brand Daikon Radish microgreens recalled due to *Listeria monocytogenes*. Recalls and Safety Alerts. https://www.inspection.gc.ca/about-the-cfia/newsroom/food-recallwarnings/complete-listing/2018-06-28/eng/1530237479767/1530237483085

[16] CFIA- Canadian Food Inspection Agency (2019) Pousses et Cie brand Mix Spicy Microgreens recalled due to Listeria monocytogenes. Recalls and Safety Alerts. https://inspection.gc.ca/food-recall-warnings-and-allergy-alerts/2019-05-22/eng/1558549526741/1558549527573

[17] Whole Foods Market (2018) Updated food recall warning—Certain Greenbelt microgreens brand microgreens recalled due to Listeria monocytogenes. https://www.wholefoodsmarket.com/content/updated-food-recall-warning-certain-greenbelt-microgreens-brand-microgreens-recalled-due

[18] Turner ER, Luo Y, Buchanan RL (2020) Microgreen nutrition, food safety, and shelf life: A review. J Food Sci 85(4):870–882. doi: 10.1111/1750-3841.15049 32144769

[19] Callejón RM, Rodríguez-Naranjo MI, Ubeda C, Hornedo-Ortega R, Garcia-Parrilla MC, Troncoso AM (2015) Reported foodborne outbreaks due to fresh produce in the United States and European Union: Trends and causes. Foodborne Pathogen Dis 12:32–38. doi: 10.1089/fpd.2014.1821 25587926

[20] Watanabe Y, Ozasa K, Mermin JH, Griffin PM, Masuda K, Imashuku S, et al. (1999) Factory outbreak of *Escherichia coli* O157:H7 infection in Japan. Emerg Infect Dis 5:424–428. doi: 10.3201/eid0503.990313 10341179 PMC2640759

[21] Buchholz U, Bernard H, Werber D, Böhmer MM, Remschmidt C, Wilking H, et al. (2011) German outbreak of *Escherichia coli* O104:H4 associated with sprouts. N Engl J Med 365:1763–1770. doi: 10.1056/NEJMoa1106482 22029753

[22] Smith A, Moorhouse E, Monaghan J, Taylor C, Singleton I (2018) Sources and survival of *Listeria monocytogenes* on fresh, leafy produce. J Appl Microbiol 125:930–942. doi: 10.1111/jam.14025 30039586

[23] Buchanan RL, Gorris LGM, Hayman MM, Jackson TC, Whiting RC (2017) A review of *Listeria monocytogenes*: An update on outbreaks, virulence, dose-response, ecology, and risk assessments. Food Control 75:1–13. doi: 10.1016/j.foodcont.2016.12.016

[24] Di Gioia F, Renna M, Santamaria P (2017a) Sprouts, Microgreens and “Baby Leaf” Vegetables. In: Yildiz F., Wiley R. (eds) Minimally processed refrigerated fruits and vegetables. *Food Engineering Series*. Springer, Boston.

[25] Reed E, Ferreira CM, Bell R, Brown EW, Zheng J (2018) Plant microbe and abiotic factors influencing *Salmonella* survival and growth on alfalfa sprouts and Swiss chard microgreens. Appl Environ Microbiol 84:e02814–e02817. doi: 10.1128/AEM.02814-17 29453267 PMC5930314

[26] Kyriacou MC, Rouphael Y, Di Gioia F, Kyratzis A, Serio F, Renna M, et al. (2016) Micro-scale vegetable production and the rise of microgreens. Trends Food Sci Technol 57:103–115. doi: 10.1016/j.tifs.2016.09.005

[27] Paradiso VM, Castellino M, Renna M, Gattullo CE, Calasso M, Terzano R, et al. (2018) Nutritional characterization and shelf-life of packaged microgreens. Food Funct 9:5629–5640. doi: 10.1039/c8fo01182f 30298894

[28] Berba KJ, Uchanski ME (2012) Postharvest physiology of microgreens. Journal of Young Investigators 24:1–5.

[29] Chandra D, Kim JG, Kim YP (2012) Changes in microbial population and quality of microgreens treated with different sanitizers and packaging films. Horticulture, Environment, and Biotechnology 53:32–40.

[30] Kou L, Luo Y, Yang T, Xiao Z, Turner ER, Lester GE, et al. (2013) Postharvest biology, quality and shelf life of buckwheat microgreens. LWT–Food Science and Technology 51:73–78. doi: 10.1016/j.lwt.2012.11.017

[31] Xiao Z, Luo Y, Lester GE, Kou L, Yang T, Wang Q (2014) Postharvest quality and shelf life of radish microgreens as impacted by storage temperature, packaging film, and chlorine wash treatment. LWT–Food Science and Technology 55:551–558. doi: 10.1016/j.lwt.2013.09.009

[32] Yadava DK, Yashpal, Vasudev S, Singh N, Saini N, Prabhu KV, et al. (2019) Indian mustard: Variety Pusa Double Zero Mustard-31 (PDZM-31). Indian Journal of Genetics and Plant Breeding 79(3):636–637.

[33] Sauer DB, Burroughs R (1986) Disinfection of seed surfaces with sodium hypochlorite. Phytopathology 76:745–749.

[34] Luo Y, McEvoy JL, Wachtel MR, Kim JG, Huang Y (2004) Package atmosphere affects postharvest biology and quality of fresh-cut cilantro leaves. HortSci 39:567–570. doi: 10.21273/HORTSCI.39.3.567

[35] Allende A, Luo Y, McEvoy JL, Artés F, Wang CY (2004) Microbial and quality changes in minimally processed baby spinach leaves stored under super atmospheric oxygen and modified atmosphere conditions. Postharvest Biol Technol 33:51–59. doi: 10.1016/j.postharvbio.2004.03.003

[36] Sharma P, Sharma A, Rasane P, Dey A, Choudhury A, Singh, et al. (2020) Optimization of a process for microgreen and fruit-based functional beverage An Acad Bras Cienc 92(3):e20190596. doi: 10.1590/0001-3765202020190596 33111819

[37] Kim JG, Luo Y, Gross KC (2004) Effect of packaging film on the quality of fresh-cut salad savoy. Postharvest Biol Technol 32:99–107. doi: 10.1016/j.postharvbio.2003.10.006

[38] Das BK, Kim JG (2010) Microbial quality and safety of fresh-cut broccoli with different sanitizers and contact times. J Microbiol Biotechnol 20(2):363–369. doi: 10.4014/jmb.0907.07009 20208442

[39] Choe U, Yu LL, Wang TTY (2018) The science behind microgreens as an exciting new food for the 21^st^ century. J Agric Food Chem 66:11519–11530. doi: 10.1021/acs.jafc.8b03096 30343573

[40] Benincasa P, Falcinelli B, Lutts S, Stagnari F, Galieni A (2019) Sprouted grains: A comprehensive review. Nutrients 11:421. doi: 10.3390/nu11020421 30781547 PMC6413227

[41] Abad M, Noguera P, Burés S (2001) National inventory of organic wastes for use as growing media for ornamental potted plant production: case study in Spain. Bioresour Technol 77:197–200. doi: 10.1016/s0960-8524(00)00152-8 11272028

[42] Di Gioia F, Mininni C, Santamaria P (2015) How to grow microgreens. In Di Gioia F., Santamaria P. (Eds.), Microgreens: Microgreens: Novel fresh and functional food to explore all the value of biodiversity (pp. 51–79). Italy: ECO-logicasrl Bari

[43] Murphy CJ, Pill WG (2010) Cultural practices to speed the growth of microgreen arugula (roquette; *Eruca vesicaria* subsp. *sativa*). J Hort Sci Biotechnol 85:171–176. doi: 10.1080/14620316.2010.11512650

[44] Moura A, Disson O, Lavina M, Thouvenot P, Huang L, Leclercq A, et al. (2019) Atypical hemolytic *Listeria innocua* isolates are virulent, albeit less than *Listeria monocytogenes*. Infect Immun 87:e00758–18. doi: 10.1128/IAI.00758-18 30670551 PMC6434133

[45] Chandra D, Choi AJ, Kim YP, Kim JG (2015) Physicochemical, microbial and sensory quality of fresh-cut red beetroots in relation to sanitization method and storage duration. Italian Journal of Food Sciences 27(2):80–92. doi: 10.14674/1120-1770/ijfs.v188

[46] Xiao Z, Bauchan G, Nichols-Russell L, Luo Y, Wang Q, Nou X (2015) Proliferation of *Escherichia coli* O157:H7 in soil-substitute and hydroponic microgreen production systems. Journal of Food Protection 78:1785–1790. doi: 10.4315/0362-028X.JFP-15-063 26408126

[47] Park HK, Kushad MM, Feng H (2013) Survival of *Escherichia coli* O157:H7 strain87-23 on arugula, kale, lettuce and mizuna microgreens, and comparison of leaf surface morphology for mature greens and microgreens. Presented at Poster session, IAFP Annual Meeting, Charlotte, NC.

[48] Di Gioia F, De Bellis P, Mininni C et al (2017b) Physicochemical, agronomical and microbiological evaluation of alternative growing media for the production of rapini (*Brassica rapa* L.) microgreens. J Sci Food Agril 97:1212–1219. doi: 10.1002/jsfa.7852 27311947

[49] Wright KM, Holden NJ (2018) Quantification and colonisation dynamics of *Escherichia coli* O157:H7 inoculation of microgreen species and plant growth substrates. International Journal of Food Microbiology 273:1–10. doi: 10.1016/j.ijfoodmicro.2018.02.025 29554556

[50] Warriner K, Ibrahim F, Dickinson M, Wright C, Waites WM (2003) Internalization of human pathogens within growing salad vegetables. Biotechnology and Genetic Engineering Reviews 20:117–136. doi: 10.1080/02648725.2003.10648040 14997849

[51] Brecht JK (1995) Physiology of ligthy processed fruits and vegetables. Hortscience, 30(1):18–22. doi: 10.21273/HORTSCI.30.1.18

[52] Watada AE, Ko NP, Minott DA (1996) Factors affecting quality of fresh-cut horticultural products. Postharvest Biology and Technology, 9(2):115–125. doi: 10.1016/S0925-5214(96)00041-5

[53] Deza-Durand KM, Petersen MA (2011) The effect of cutting direction on aroma compounds and respiration rate of fresh-cut iceberg lettuce (*Lactuca sativa* L.). Postharvest Biol Technol 61(1):83–90. doi: 10.1016/j.postharvbio.2011.02.011

[54] Wang H, Feng H, Luo Y (2004) Microbial reduction and storage quality of fresh-cut cilantro washed with acidic electrolyzed water and aqueous ozone. Food Res Intl 37:949–956. doi: 10.1016/j.foodres.2004.06.004

[55] USFDA- U.S. Food and Drug Administration Center for Food Safety and Applied Nutrition [US FDACFSAN]. (1999). Guidance for Industry: Reducing microbial food safety hazards for sprouted seeds. Federal Register 64(207):57893–57902. https://www.govinfo.gov/content/pkg/FR-1999-10-27/pdf/99-28016.pdf

[56] Mir SA, Shah MA, Mir MM (2017) Microgreens: Production, shelf life and bioactive components. Critical Reviews in Food Science and Nutrition 57(12):2730–2736. doi: 10.1080/10408398.2016.1144557 26857557

