# Supplementary figures and images for: Yield optimization, microbial load analysis, and sensory evaluation of mungbean (Vigna radiata L.), lentil (Lens culinaris subsp. culinaris), and Indian mustard (Brassica juncea L.) microgreens grown under greenhouse conditions
Source: PLoS One. 2022 May 24;17(5):e0268085. doi: 10.1371/journal.pone.0268085 (PMC9128967; doi:10.1371/journal.pone.0268085)

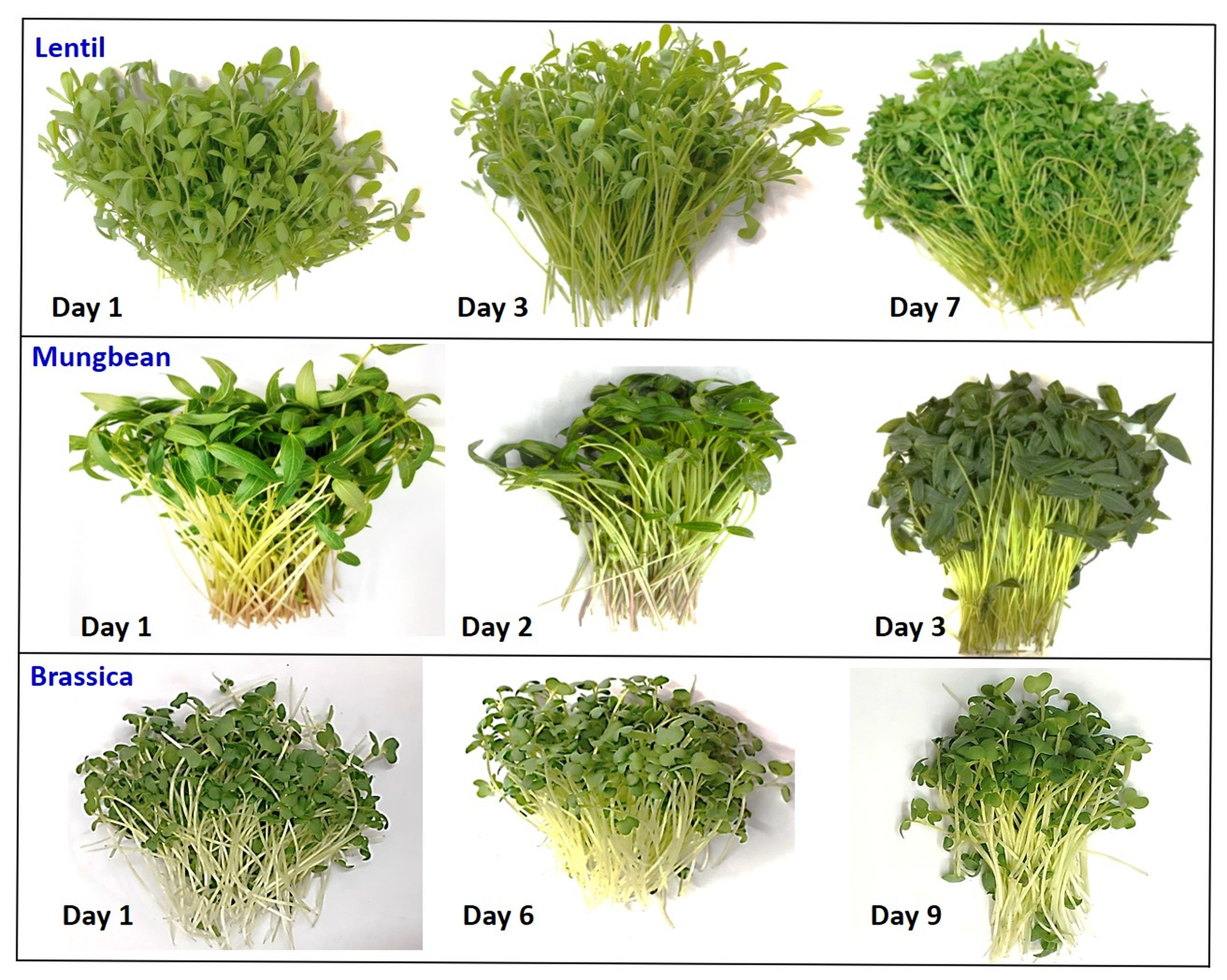

Supplement: S1 Fig — (TIF) [file pone.0268085.s001.tif]

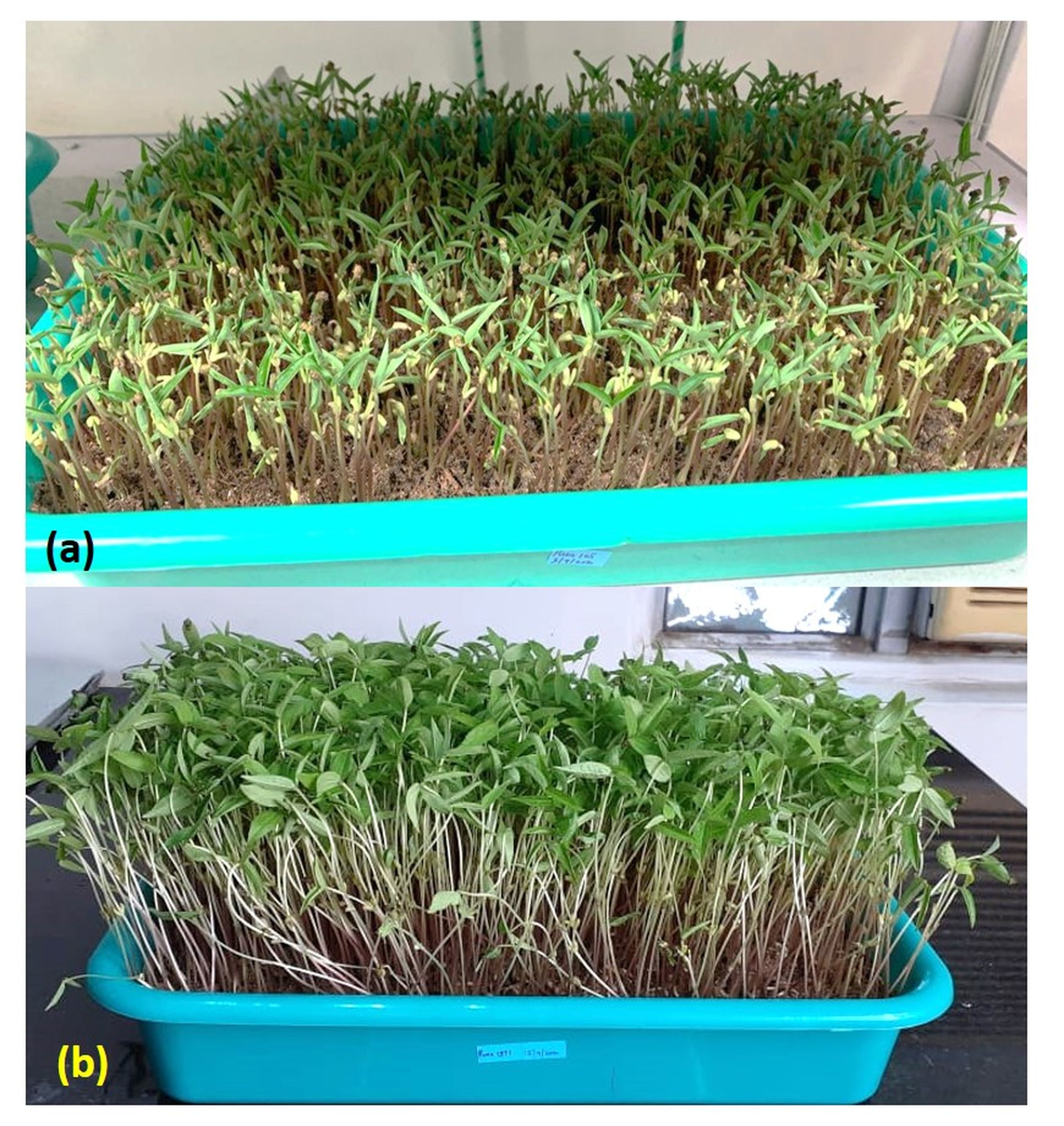

Supplement: S2 Fig — (TIF) [file pone.0268085.s002.tif]
